# Perspectives on digital therapeutic prescribing: a qualitative study among German psychological psychotherapists

**DOI:** 10.3389/fdgth.2026.1656614

**Published:** 2026-02-09

**Authors:** Karin Panitz, Charlotte Wagenaar, Fatma Sahan, Nadja Kairies-Schwarz, Lisa Guthardt, Jessica T. Bau, Adrian Loerbroks, Markus Vomhof, Maximilian Zinn, Claudia R. Pischke, Peter Angerer, Jennifer Apolinário-Hagen

**Affiliations:** 1 Institute for Occupational, Social and Environmental Medicine, Faculty of Medicine, Heinrich Heine University Düsseldorf and University Hospital Düsseldorf, Düsseldorf, Germany; 2 Center for Digital Medicine, Heinrich Heine University Düsseldorf, Düsseldorf, Germany; 3 Institute for Health Services Research and Health Economics, Faculty of Medicine, Heinrich Heine University Düsseldorf and University Hospital Düsseldorf, Düsseldorf, Germany; 4 Institute for Health Services Research and Health Economics, German Diabetes Center, Leibniz Center for Diabetes Research at the Heinrich Heine University Düsseldorf, Düsseldorf, Germany; 5 Institute of Medical Sociology, Faculty of Medicine, Heinrich Heine University Düsseldorf and University Hospital Düsseldorf, Düsseldorf, Germany

**Keywords:** attitude, digital health, information literacy, mental health, mobile applications, psychotherapists, qualitative research, telemedicine

## Abstract

**Background:**

Since 2020, psychological psychotherapists in Germany can issue CE-certified prescription digital therapeutics (German *Digitale Gesundheitsanwendungen*, DiGA) at the expense of statutory health insurance. Although DiGA must adhere to rigorous requirements (e.g., efficacy, data security) and are free of charge for statutorily insured patients, various barriers hinder their regular prescription in healthcare. However, little is known about facilitating and hindering factors influencing the acceptance and professional use of DiGA among psychological psychotherapists in Germany, who are authorized to issue prescriptions since the introduction of DiGA. This study thus aims to understand German psychological psychotherapists' perspectives toward prescribing DiGA.

**Methods:**

A qualitative study design was used to explore prior knowledge of, experience with, and attitudes toward prescribing DiGA, as well as information needs and wishes. Two researchers conducted semi-structured interviews in July and August 2024 with 13 licensed German psychological psychotherapists. Audio recordings of the interviews were transcribed verbatim and analyzed using deductive and inductive coding according to qualitative content analysis.

**Results:**

Psychotherapists indicated moderate knowledge about DiGA, primarily gained through professional exchanges and driven by personal interest. While psychotherapists differed in their experience with prescribing DiGA, the reported need for more information on several aspects was a shared matter. Regarding the attitudes toward prescribing DiGA, perceived advantages included bridging waiting times, enhancing therapy support, and improving aftercare. Subjective barriers included a lack of established prescribing routines (unlike physicians) or skepticism about managing patients' crises effectively while using DiGA. Wishes pertaining to the informational content of DiGA included an evidence base, data privacy, or prescription guidelines. Psychotherapists varied broadly in their preferred methods of information delivery (e.g., in sources such as collegial exchange or media channels, such as online workshops).

**Discussion:**

The findings indicate that despite a positive mindset toward DiGA, the absence of routine practice hinders its widespread adoption among psychotherapists in Germany. Increasing knowledge about DiGA may lead to greater adoption of prescribing. This qualitative formative research will form the basis for further research to evaluate and optimize information strategies regarding digital therapeutics. Future research should focus on the influence of tailored information on actual prescriptions.

## 1 Introduction

Mental illness is a global problem and one of the most significant health concerns (1, 2). By the end of 2024, nearly one in three adult respondents from 16 countries across Europe, Asia, and the United States reported currently experiencing mental health issues (3, 4). Additionally, more than a quarter of employees indicated that they had taken leave for mental health reasons during the same year. Further, the economic burden of mental disorders was associated with losses of over 3 trillion US dollars (around 2,6 trillion euros) in 2019 (5). For flexible, low-threshold services, digital mental health interventions (DMHIs) offer new opportunities for therapy in various medical fields. DMHIs can improve treatment quality, sustainability, and patients' health literacy (6). In addition, by incorporating DMHIs, psychological interventions can be transformed into standalone or blended care model formats, combining face-to-face and online delivery (7). A specific form of DMHIs are digital therapeutics (DTx) that deliver evidence-based medical intervention, for example, to treat various diseases, through different technologies (i.e., smartphones, tablets) (8, 9).

Germany was the first country worldwide to integrate prescription of CE-certified DTx (German *Digitale Gesundheitsanwendungen*, DiGA) into standard care, allowing reimbursement through statutory health insurance (6, 10–12). This was enabled by the passing of the *Digital Healthcare Act* on December 18, 2019 (13) and related regulations aiming to accelerate the digitalization of healthcare, such as the Digital Law (German *Digital-Gesetz*, Digi-G; commencement on March 26, 2024) (14). DiGA must complete an assessment by the German Federal Institute for Drugs and Medical Devices (German *Bundesinstitut für Arzneinmittel und Medizinprodukte*, BfArM) to be added to the DiGA directory to ensure the possibility of prescription and cost coverage (15, 16).

To optimize the adoption of such novel options among prescribers, awareness needs to be raised among therapists, patients, and the general public (17, 18). Since people access information on mental health primarily through healthcare professionals, including psychotherapists (3), it appears promising to raise therapists' awareness of DMHIs, especially DiGA. In recent years, a variety of DMHIs have been introduced to improve the dissemination of interventions for the treatment of common mental disorders on a large scale (19). Several meta-analyses report DMHIs based on cognitive behavioral therapy (CBT) to be effective in reducing the symptoms of mood and anxiety disorders (20–22). Reviews and analyses on DiGA in mental health report outcomes with relatively large effect sizes (Cohen's *d* = 0,16–1,79) (23, 24). Accordingly, DiGA have gradually been included in Germany's top-level evidence-based clinical “S3 Guidelines”, developed by expert consensus (25, 26), i.e., for the treatment of unipolar depression or anxiety disorders (27).

By the end of December 2025, 58 DiGA were listed in the DiGA directory, 30 of which addressed mental disorders. The uptake of DiGA increases, and 30% of all redeemed activation codes relate to DiGA, addressing mental illnesses (27, 28). About 11% of those DiGA (i.e., *deprexis, somnio, or Selfapy Depression*) are prescribed by psychological psychotherapists, around 50% by general practitioners (GPs), and about 16% by psychiatrists (28). Recent studies, involving various healthcare providers, primarily GPs, specialist doctors, or other clinicians, with psychological psychotherapists rarely represented, identified several barriers that hinder the prescribing of DiGA. These restraints include skepticism regarding the usefulness of DMHIs, data security, privacy, confidentiality, lack of knowledge, and concerns about patient adherence (10, 29–31). Additional obstacles to the broad application of DiGA are organizational problems, bureaucratic activation, missing interoperability with the practice software, the lack of testing options, and other technical constraints. Hence, information could be the key to overcoming barriers to using DMHIs professionally.

In our study, we focused on psychological psychotherapists as they represent the primary target and key multipliers for adopting DMHIs like DiGA for mental health. Since 1999, psychotherapy has required at least three years of postgraduate training for psychologists or physicians (32). Following a 2019 reform, becoming a psychological psychotherapist now involves five years of university study and five years of training (33). The programs feature comprehensive theoretical components focused on traditional methods, yet they do not incorporate telemedicine or digital skills as essential elements (32, 33). Since DiGA was integrated into standard care in Germany in 2020, it is for the first time that psychological psychotherapists are allowed to prescribe DiGA, equivalent to physicians.

However, little is known about facilitating and hindering factors influencing the acceptance and professional use of DiGA among psychological psychotherapists, such as prior knowledge of, experience with, and attitudes toward prescribing DiGA, as well as information needs. To find out which information wishes regarding DMHIs, especially DiGA for mental health, are reported by licensed psychological psychotherapists who are currently working in primary care settings in Germany, we aimed to answer the following research questions (RQ):

(1) What do psychotherapists in Germany already know about DiGA?
(2) Which experiences do psychotherapists have with recommending or prescribing DiGA?
(3) Which attitudes do psychotherapists have toward recommending or prescribing DiGA?
(4) What do psychotherapists want to know about DiGA (e.g., content of information)?
(5) How do psychotherapists want to be informed about DiGA (e.g., by which sources and via which media channels)?

To address RQ2 and RQ3, we examined individual factors influencing DiGA adoption by asking psychotherapists about both recommendations and prescriptions, irrespective of prior DiGA experience. In our study, a prescription is a documented, reimbursable order issued by an authorized provider. In contrast, a recommendation is a professional, non-binding suggestion that may prompt patients to request the DiGA directly from their insurer. Although the two actions differ legally, we present therapists' responses for both because many German psychological psychotherapists are less familiar with the formal prescription process than physicians (34).

## 2 Materials and methods

This qualitative study is a sub-study of a broader exploratory sequential mixed-methods study funded by the German Research Foundation (grant no. 528399867), exploring information needs regarding prescriptions of DiGA among healthcare professionals, such as psychotherapists, GPs, and occupational physicians. Results concerning surveyed GPs are reported in a separate publication (35). Further results and qualitative data on identified attributes and levels for a discrete-choice experiment (DCE) to elicit information preferences will be reported elsewhere.

### 2.1 Study design

A qualitative interview study was conducted to gain new insights into the perspectives of psychological psychotherapists regarding their needs, attitudes, and wishes for information about DiGA for mental health purposes in their daily practice. As evidence remains sparse, we chose semi-structured interviews, which facilitate an in-depth understanding of underexplored topics (36, 37). This applies to the needs assessment and wishes regarding information strategies on DiGA. To ensure transparent and complete reporting, we adhered to the Consolidated Criteria for Reporting Qualitative Research (COREQ, see Supplementary Table S1) (38). The Institutional Review Board of the Medical Faculty of the Heinrich Heine University (HHU) Düsseldorf provided ethical approval of the interview study (number 2023-2338) on February 7, 2023. We preregistered the formative substudy of the research project at the Open Science Framework website on June 10, 2024 (https://osf.io/uj8gt).

### 2.2 Recruitment

Individuals were eligible to participate if they were registered as licensed psychological psychotherapists in Germany with the legal option to prescribe DiGA for mental disorders at the expense of statutory health insurance companies (i.e., authorization from the Health Insurance Company's Association). Another inclusion criterion was fluency in German, the language for the interviews. Participants were recruited via social media (e.g., Instagram, LinkedIn) and German professional associations (e.g., the Federal Association of Psychotherapists Chamber, the Federal Association of Contract Psychotherapists, the German Psychotherapists Network). The recruitment material consisted of postings, flyers, emails, and a short video. It contained brief information about the study and its inclusion criteria, along with a link to sign up. Psychotherapists who expressed interest in participating were provided with detailed study information, the informed consent form, and a brief background questionnaire with 13 items on sociodemographic details as well as on DiGA experience (see Supplementary Table S2). A convenience sampling strategy was applied. Participants received a compensation of 100 euros. Of 39 interested therapists, 10 were excluded, not meeting the inclusion criteria. After reaching a point at which it could no longer be assumed that further interviews would provide new insights (i.e., theoretical saturation), 16 licensed interested psychotherapists meeting the inclusion criteria were declined (see Supplementary Figure S1).

### 2.3 Data collection

Interviews were conducted online using the videoconferencing software Webex (Cisco Systems, CA, USA), with access provided through the institution (HHU) for university staff to facilitate recruitment across Germany. Participants were advised not to enter personal data into Webex and could decline using the webcam. The video was not recorded; instead, audio files were recorded using a voice recorder without an internet connection. Participants could also choose to be interviewed by telephone (via the University Hospital Düsseldorf's conventional telephone network); however, this option was not utilized. Pilot interviews with three female psychologists were conducted to test the technical implementation and the content-related process (39–41). The interview guide (see Supplementary Table S3) was developed by JAH, grounded on empirical research (42), and discussed within the study team. It was slightly adjusted after the pretests and the first interviews by KP and CW. Online interviews were conducted from July 5 to August 16, 2024, by KP (*n* = 10) and CW (*n* = 3), held in German. Both interviewers, female doctoral students, were trained in qualitative interview methods beforehand. The interviewers had no contact with the participants before recruitment. They introduced themselves as a psychologist (KP) and a medical graduate (CW) before the interview started, briefly explaining their personal interest in DMHIs. Only the participant and the interviewer were present during the online interview. Interviews were audio-recorded, pseudonymized, and transcribed verbatim using artificial intelligence with manual control compliant with general data protection regulations. The transcriptions were revised and checked for accuracy by the respective interviewers before being analyzed. Participants were not asked to provide feedback on the transcripts. However, until anonymization, they had the opportunity to review their transcripts (as documented in the study information). After analyzing the transcriptions, all audio records were deleted. Likewise, the interviewers' field notes were shredded. After writing the results, the pseudonymization key was deleted, and transcriptions were anonymized.

Interviews were conducted until theoretical saturation (i.e., the point at which no further information gain is to be expected) (43, 44), which was first discussed after nine interviews and considered fully achieved after thirteen interviews.

### 2.4 Data analysis

Building on Kuckartz and Rädiker (45), a content-structured qualitative content analysis with an inductive-deductive approach was conducted. The transcripts were coded and analyzed in several steps. Based on the semi-structured interview guide, KP and CW developed the main categories, which were discussed with the principal investigator (JAH) of the mixed-methods study, an expert in the field of the determinants of the acceptance and use of DMHIs. They then coded three interviews independently to develop subcategories inductively, which the senior researchers JAH and AL, with thematic and methodological expertise, reviewed. After another round of independent coding of two additional interviews, the doctoral students KP and CW iteratively adjusted the coding system. JAH and FS subsequently reviewed the coding system, suggested refinements, and then approved this final coding system, after which KP analyzed the interviews. The final coding system was further discussed with other project team members, including experienced researchers on provider research (NKS, MV, MZ) and qualitative research (LG, JB). The research team members involved in the process come from diverse training and academic backgrounds, including psychology (KP, FS, CP, JAH), medicine (CW, PA), health economics (NKS, MV, MZ), health services research (AL), public health (LG, JB, AL, CP), epidemiology (AL, CP), occupational medicine in health sciences (AL, JAH), medical sociology (JB, CP), and literary translation (LG). This ensures intersubjective transparency, replicability, and discriminatory power of categories and levels. The coding scheme contained three levels, with deductive and inductive categories (Supplementary Table S4 shows, for clarity, two of the three levels with anchor examples).

## 3 Results

The sociodemographic characteristics of the study participants and their awareness of DiGA for mental health purposes are reported quantitatively. Qualitative data regarding participants' prior knowledge about DiGA (RQ1), their experience with recommending and prescribing DiGA (RQ2), their attitudes toward recommending or prescribing DiGA (RQ3), as well as their information requests (RQ4) and the preferred delivery mode (RQ5) are presented due to the coding scheme. Findings were derived from the data and supported with quotes, marked with the abbreviation PT for psychotherapist, the participant's number, and gender. Main topics are clearly outlined with paragraph titles. Illustrative quotes were translated from German to English by the first author (KP) and a professional translator (LG).

### 3.1 Sample characteristics

In total, *n* = 13 online interviews with psychological psychotherapists were conducted and analyzed (i.e., no dropouts), lasting 32.53 min on average (range: 25.19–43.10 min). As shown in Table 1, the mean age of the participants was 38 years (*M* = 38.08, *SD* = 5.06), and 69.2% (*n* = 9) identified as women.

**Table 1:** Participants (*N* = 13) self-reported sample characteristics based on a brief questionnaire.

Variable	Specification
Demographics	
Age in years: *M* (*SD*), min-max, median	38.08 (5.06), 30–48, 38.00
Female: *n* (%)	9 (69.2)
Years of practice: *M* (*SD*), min-max, median	11.31 (4.9), 4–21, 10.00
Education (specialization, therapy method)	
Behavioral therapy for adults: *n* (%)	9 (69.2)
Depth psychology: *n* (%)	2 (15.4)
Behavioral therapy for children/adolescents: *n* (%)	2 (15.4)
Location in Germany (federal states, type of location)	
Berlin: *n* (%)	1 (7.7)
Bavaria: *n* (%)	5 (38.5)
Hamburg and Lower Saxony: *n* (%)	1 (7.7)
Lower Saxony: *n* (%)	3 (23.1)
North Rhine-Westphalia: *n* (%)	3 (23.1)
(more) rural: *n* (%)	4 (30.8)
(more) urban: *n* (%)	8 (61.5)
Both, (more) rural and (more) urban: *n* (%)	1 (7.7)
Statutory health insurance license^a^	
Full insurance panel license: *n* (%)	7 (53.9)
Half insurance panel license: *n* (%)	2 (15.4)
Insurance panel license, part time: *n* (%)	1 (7.7)
No insurance panel license: *n* (%)	3 (23.1)

a In Germany, a statutory health insurance license allows psychotherapists to treat patients covered by the public health insurance system and bill insurers directly. These licenses, known as “*Kassensitze*” (English *insurance panel licenses*), are limited in number, regulated to meet regional healthcare needs, and can be acquired half-time to only have a “half supply mandate” (https://www.kbv.de/html/bedarfsplanung.php). Certain circumstances allow employed therapists to work in a licensed practice without owning a license.

### 3.2 Prior knowledge about DiGA

*Self-reported awareness.* In the pre-interview questionnaire, all participants reported having heard of DiGA for mental health purposes or DMHIs. Supplementary Table S5 provides detailed information regarding awareness of DiGA for mental health or DMHIs.

*Association with the term.* Participants linked DMHIs or DiGA primarily to apps for smartphones, digital-supported therapy, and named some DiGA. They associated techniques across various medical treatment areas, including respiratory issues, mental health, and smoking cessation. From their perspective, it reflects the progressive integration of new media.

*Current state of knowledge.* Participants provided detailed insights into their understanding of DiGA. Most described their knowledge as fairly moderate, often citing a perceived need to be adequately informed to prescribe and guide the responsible use of DiGA. Some participants indicated they were well-informed, typically through part-time research activities or involvement in a collaborative developmental process. Conversely, some participants felt poorly informed, indicating they only knew the mere existence of DiGA or had never worked with them*.*

*Previous information channels.* According to how they obtained their current knowledge, some participants reported having received education on DMHIs or DiGA during their *studies* or in their *psychotherapeutic training*. Other participants noted that their university programs included relevant content, and only one mentioned having attended specific topic blocks focused on online therapy. Some participants engaged in their *own inquiry*, driven by intrinsic motivation, personal interest, or a desire to learn more about DiGA:

“I feel moderately informed, because I was actually intrinsically motivated. I”ve tackled this myself, got some test accounts in my free time to find out what this is really about, so that I can prescribe it without hesitation.” (PT6, female)

Participants mentioned having already experienced *collegial exchanges*, such as through quality circles or intervision groups [collegial supervision, i.e., case discussions among colleagues], but the extent of information gained varied among them. Information from *professional associations* or the Chamber of Psychotherapists (Association of Statutory Health Insurance Physicians) was rarely cited as a source of information. Some participants noted receiving information through advertisements and the *providers*' websites, which offered them comprehensive details about DiGA.

### 3.3 Experiences with recommending or prescribing DiGA

*Previous prescription practice (behavior).* In the pre-interview questionnaire, over 60% of participants indicated having prescribed a DiGA at least once, and 77% had given advice on DiGA or DMHIs. Of those participants who had already been prescribed DiGA, one admitted to having done it only selectively. Few mentioned having recommended DiGA several times and reported that it was not really accepted by their patients.

*Effort and practicability of the prescription process.* Participants shared varying experiences regarding the effort required to recommend or prescribe DiGA. They reported needing between 2 and 30 min to complete the prescription process, partly including patient education. Some mentioned spending additional time beforehand to familiarize themselves with the process, with one interviewee indicating that this took 2–4 h. One participant noted referring patients to a general practitioner because the relevant feature had not yet been integrated into their practice software. It was stated that the amount of effort required depended on everyone's prior knowledge of the process:

“Well, before my first DiGA prescription, I had to order the prescribing pad, because, as a psychological psychotherapist, I naturally did not have that in my practice. […] It would be great to have certain forms or some kind of assistance so that it's less work. And if you’re not doing it for the first time, it takes about 5 to 10 min, I would say. I think that's realistic.” (PT8, male)

*Feedback from patients and observed usage behavior of patients.* The responses regarding feedback or prior use by patients varied significantly. Participants reported having little to no feedback; for example, they did not ask about it by default, prescribed it for outsourcing purposes, or had not seen the patient since then. Others received positive feedback, noting that the presentation was engaging, well-structured, and effectively conveyed information. Additionally, participants mentioned receiving negative feedback from patients, indicating that the setup was complicated, personalization was insufficient, demands were too high, or patients did not use the DiGA after downloading it:

“So, I prescribed app X [app addressing, i.e., Agoraphobia, Panic disorder] twice, and neither of them used it. One of them didn't even redeem it, and I don't know about the other one. I haven't seen him again. He was strongly impaired, so he might not have been able to manage it.” (PT12, female)

One participant suggested an option to familiarize patients with DiGA:

“You should make it appealing, so to speak. Patients should have the opportunity to use it in the practices. They could try it out in the waiting room on a tablet, for example, in a safe way that complies with data protection regulations. I think this might be a good way to establish initial contact.” (PT1, male)

### 3.4 Attitudes toward recommending or prescribing DiGA

We specifically asked participants about areas in which they believed they could improve their understanding of DiGA, and factors motivating them to seek more information or boost the prescription of DiGA. Additionally, we inquired about perceived benefits and barriers related to prescribing DiGA.

*Need for information improvement.* Participants suggested that enhancing the dissemination of information could focus on technical aspects. They identified specific gaps in their ability to prescribe DiGA, noting that a comprehensive guideline would be beneficial. In addition, they expressed uncertainty about when to issue prescriptions and how to integrate this process into the routine practice effectively.

*Incentives to look for more information or prescribe DiGA.* Participants acknowledged that numerous obligations and limited time were key barriers to prescribing DiGA. They noted that one advantage of engaging with DiGA would be improved compensation, such as financial remuneration, Continuing Medical Education (CME) points, and free training. They also mentioned that seeing patients succeed through a DiGA would be an additional incentive.

*Perceived benefits of DiGA.* According to the participants, the advantages of DiGA include bridging waiting periods and serving as a supplement or partial substitute for face-to-face therapy. This can be particularly beneficial for improving initial motivation, especially in rural areas, and for outsourcing psychoeducation, knowledge transfer, or exposure therapy. From their perspective, DiGA also aids in aftercare or relapse prevention. One participant noted that DiGA could facilitate patient access, especially for those who may feel hesitant or face difficulties seeking therapy. Others mentioned that DiGA could serve as support after basic care, highlighting the usefulness of special journal functions.

*Perceived barriers of DiGA.* Participants highlighted several barriers to recommending or prescribing DiGA. It was noted that, despite having a positive mindset, the lack of established routines seemed to hinder their use. Few participants expressed skepticism about using DiGA as a substitute or temporary alternative for face-to-face therapy, arguing that it might lead to an underestimation of psychotherapy's effectiveness, prevent patients from beginning person therapy, and may not adequately address complex disorders. Another concern raised was the risk of overwhelming patients, particularly due to unsuitable applications or insufficient personal support. Some participants worried that transitioning therapy to the digital realm could lead to the elimination of their current practices due to competition or lower costs. There were also concerns regarding managing upcoming patient's crises effectively:

“I still don’t know about questions on how to handle crises, how to handle suicidal tendencies, because these are major issues. What is the impact of the therapeutic alliance? How can you appropriately address this in digital therapeutics?” (PT9, female)

Participants also expressed concerns about potentially facing recourse claims^1^ for their DiGA prescriptions.

#### 3.4.1 Comparison of prescribers' and non-prescribers' attitudes toward DiGA

To better understand how attitudes toward prescribing differ based on experience, we compared the statements of experienced prescribers (*n* = 8) with those of non-prescribers (*n* = 5). Supplementary Table S6 presents this comparison. Experienced prescribers identified more distinct benefits and reported fewer barriers compared to non-prescribers. This difference was less noticeable regarding the need for information improvement or incentives to seek for more information or prescribe DiGA.

### 3.5 Information wishes about DiGA

To better understand their information needs and wishes, participants were asked about the essential content required for making informed decisions regarding the prescription of DiGA for mental health purposes.

*Evidence and efficacy.* All participants preferred obtaining information about the evidence and efficacy, with varying levels of detail. This information should include an updated meta-analysis addressing evidence and be freely and easily accessible in the register. Some participants felt confident that the admission regulations of DiGA had been thoroughly tested.

*Data protection.* Participants expressed that information on data privacy was a priority for them. They wanted to be informed about server locations and types of data processing. However, other participants indicated that they trusted the approval regulations of DiGA.

*Cost information.* Participants were interested in information about costs for various reasons. However, they emphasized the need for transparency regarding this issue. They wanted to communicate the value of the therapy to patients, assess its potential impact on the healthcare system, compare different DiGA, or address health and professional policy reasons:

“I think information about the costs of these health applications is interesting, because this is also some sort of indication of what the provider charges for an app. If there's an app that's twice the price, I’d naturally expect it to be more functional and have a greater scope.” (PT8, male)

*User-friendliness.* Participants highlighted the importance of strong usability, including availability across various platforms, language options, geriatric psychiatric aspects, accessibility settings for individuals with (learning) disabilities, and user numbers to be able to evaluate adherence.

*App structure and design.* Understanding the specific structures was essential for making informed prescribing decisions for participants. They wanted to know about various key elements, such as the availability of chat features, components for activity development, cognitive restructuring, duration of use, length of the program, individualization options (e.g., adjustment of the speed), own possible contact person (e.g., psychologist), push notifications, and reminders. The information about therapeutic principles (e.g., manual-based, in accordance with guidelines) seemed crucial to ensure alignment with the therapeutic approach or determine how the tool could be utilized and integrated into their therapy practice:

“Of course, I’d like to know, how independently can a patient really work with it? Or is it more of a tool to support the actual therapy?” (PT4, male)

Participants stated it would be helpful for them to receive examples of the apps, such as screenshots of certain features.

*Other prescription-relevant factors.* Participants mentioned additional relevant factors to make responsible recommendations or prescriptions, such as indications, contraindications, or specific socio-demographic information of patients. It was proposed that information could be similar to the yellow list of the pharmaceutical industry. Further, it would be helpful for some participants if this information were presented in a clear, tabular format that compares different applications, highlighting their advantages and disadvantages.

*Prescription modalities.* Participants suggested adaptations and adding information to the existing prescription procedure, such as implementation in the clinic or practice software and prescription forms:

“I think the prescription pads or forms make it a lot easier for many clinicians. Things would be so much easier if you didn't need to take care of this yourself. I think that's one of the greatest challenges. The more automated the process is, the less the therapists must do themselves. Integrating it into the clinic software would also be an option, so being able to do it at the touch of a button, more or less.” (PT8, male)

*Updates on certain DiGA.* Participants suggested that receiving information about the latest updates could lead to a reassessment of a DiGA that was previously deemed uninteresting.

*Developer/provider background.* To evaluate the professional background effectively, participants found it important to obtain detailed information about the provider or developer team of certain DiGA.

### 3.6 Information wishes regarding delivery mode

*Information sources.* Participants identified the German *Chamber of Psychotherapists, the Association of Statutory Health Insurance Physicians*, or the various *professional associations* as reliable sources of information. From their perspective, these organizations serve as the primary contact points, providing all essential information relevant to their work and therapy. Additionally, respondents emphasized the importance of *collegial exchange*, which includes insights from experienced colleagues, professionals, research, and established guidelines. Furthermore, participants expressed interest in receiving information from *legislators* (e.g., the German Federal Ministry of Health, BfArM, DiGA directory), as these entities are responsible for quality assurance. Some participants preferred obtaining information from *training or education institutes*, seeking neutral perspectives, and receiving CME points. Other participants found *testimonials from patients* credible, particularly if they came from their patients or relatives. However, participants viewed anonymous evaluations skeptically, as they could be fabricated for promotional purposes. *Manufacturers*' websites were generally regarded as good sources for insider information, while aggressive advertising emails were viewed negatively, especially if they became overwhelming. Participants thought that *health insurance funds and other payers* are not a completely trustworthy source of information, as they might have a vested interest:

“I have to differentiate between health insurance companies [as an information source]. On the one hand, I would trust them to have checked it in detail. With regard to health economics, on the other hand, I would also assume that they are quite interested in prescribing it, because it is certainly the cheaper alternative to in-person therapy, for example.” (PT13, female)

Other sources mentioned included *(public) media*, *practice management software*, and *social networks* such as Instagram and LinkedIn.

*Media channels.* Various access forms were mentioned, including *virtual* options and *print* materials such as specialist journals, brochures, and manuals. Additionally, digital and postal *newsletters* or *training courses* (digital and face-to-face) and *online searches* in various portals and search engines were mentioned. Participants expressed a desire to get test access for trying out the app to have a better understanding:

“Oh, yes, with DiGA, it also makes sense to watch a demonstration or get a test access. Just to get an impression of the usability and how you're guided through the program, and to see what components are most appealing, to find out if I would recommend it to a patient or not.” (PT4, male)

### 3.7 Additional insights

*Willingness to invest in information acquisition.* For an initial *search*, participants were willing to spend 5–10 min for a quick overview or first exploration of the topic. If the search had a specific purpose, this time could extend up to 2–3 h, depending on prior knowledge and experience. For *further training*, participants were ready to invest from 1 h up to 2 days, depending on their actual workload and personal circumstances, if they saw added value for their work, and the content was rich. Generally, participants were not inclined to pay for further training on DiGA, given that the system was, among other things, designed to alleviate their workload. However, depending on the training's depth, duration, and delivery mode (e.g., in-person, virtual, live, or recorded), they might be willing to invest between 50 and 350 euros.

*Attitudes toward artificial intelligence (AI).* We also asked participants for their opinion on receiving information about the latest developments concerning AI and specific wishes on how to be informed most effectively. Participants emphasized the importance of AI in their field, stating that it might ease everyday practice. Concerns were raised regarding, e.g., data protection or patient safety. The increasing relevance also seems to arise from patients addressing this issue in therapy:

“Oh yes, I think it [AI] is very important, because […] it can be used to facilitate everyday life. AI-supported tools might somehow help write reports, for example. On the other hand, it can also help us understand what's currently going on in the world. AI is becoming more important, so people will inevitably bring up the topic in therapy. And I think it's very important to simply know what they're talking about.” (PT5, female)

Participants preferred digital information, free of charge (e.g., through email), that sheds light on various aspects of AI or multi-sectional articles to show the latest developments, supported, for example, by the BfArM, the Chamber of Psychotherapists, or the German Federal Ministry of Health.

## 4 Discussion

This study aimed to evaluate the facilitating and hindering factors influencing the prescription of DiGA among psychological psychotherapists in Germany. When comparing the results presented here to previous studies, it is important to note that existing research primarily involves experienced physicians when issuing prescriptions, even though prescribing DiGA is a relatively new practice for psychological psychotherapists in Germany.

### 4.1 Main findings and comparison with prior work

#### 4.1.1 Prior knowledge about DiGA for professional use (RQ1)

Our findings indicated that the surveyed psychotherapists were aware of DiGA for therapeutic purposes. They had already advised their patients on using DiGA for mental health purposes or non-prescription DMHIs, or had already prescribed DiGA. Most psychotherapists expressed being informed moderately, which they attributed to professional exchanges and self-directed interest. Prior studies also including therapists in clinical training report lower proportions (30, 46, 47), but it is to be expected that knowledge increases over time.

#### 4.1.2 Experience with recommending or prescribing DiGA (RQ2)

Two-thirds of our participants reported prescribing a DiGA at least once. Other quantitative studies revealed lower rates, with only one-quarter of surveyed psychotherapists recommending DMHIs, including DiGA (30), and one-third of 2022 surveyed psychologists having experience with prescribing DiGA (48). In our study, the time taken to complete prescriptions varied from 5 to 30 min after some practice. This aligns with the quantitative findings from Weitzel et al. (30), in their study, three-quarters of the psychotherapists rated their recommendation efforts as rather low. A rapid review involving a diverse group of health care professionals indicated that the effort required for prescription was high (49). Our findings suggest that developing routines may help reduce the time required for prescriptions. According to patients' feedback on DiGA, our findings revealed inconsistent and varied responses. Some see patients' success as a good motivation to prescribe, and others do not think having patients' testimonials is necessary. In this special context, it would be interesting to further explore whether negative patient feedback can be viewed as a hindering factor, deterring therapists from prescribing DiGA (30).

#### 4.1.3 Attitudes toward recommending or prescribing DiGA (RQ3)

Attitudes among participants were overall positive, but they also mentioned several drivers and barriers to prescribing DiGA. For example, the surveyed psychotherapists noted that a guideline with important prescribing regulations and additional technical information would enhance their ability to prescribe DiGA. Earlier studies also report a lack of guidelines and complicated prescription processes (29, 49), suggesting that developing best practices may ease the adoption of digital technologies (50).

In line with Berardi et al.'s findings, a high workload and limited time were reported to be significant barriers to seeking information about or prescribing DiGA (29). Psychotherapists in our study indicated they would be more likely to engage with DiGA if they received financial compensation, CME points, or free training. This aligns with other studies that report economic incentives, such as payments, and non-economic incentives, such as awards, are effective motivators for increasing the demand for digital technologies. Furthermore, other research noted a lack of financial incentives as a concern (51). Therefore, strategies that address these issues should be considered and further investigated to optimize the adoption of DiGA.

Our study revealed the following benefits and opportunities of DiGA, confirming previous findings: Bridging waiting periods (30), supporting face-to-face therapy (29, 30, 49), enhancing patient motivation (49), improving aftercare (29, 30), relapse prevention, and facilitating access to treatment (29) through features like journaling (49).

We found that the challenges and perceived risks associated with prescribing DiGA are in line with findings from previous research. There's skepticism regarding DiGA's effectiveness for complex mental disorders, which highlights the importance of robust risk management and treatment options for severe or acute mental health conditions (29). According to our participants, DiGA may deter patients from starting face-to-face therapy or overwhelm patients with unsuitable options, confirmed by Kreuzenbeck et al. (49), who noted that DiGA could potentially encourage patients in self-diagnosis and self-treatment or be too complicated. According to the suggestions of Berardi et al. (29), effective crisis management concerns can be alleviated by implementing safety protocols, especially in case of an emergency. The challenges and perceived risks go beyond individual uncertainty and raise fundamental questions of professional responsibility and clinical governance, especially for crisis management and complex disorders. In our study, some participants noted that it is more important to consider which patients are suitable for DiGA than which DiGA is suitable for specific patients. They argued that in some cases, therapeutic support is essential. Education and training for therapists, who determine the medical necessity of DiGA, are therefore required to support informed decision-making. Above that, robust oversight mechanisms and explicit liability arrangements are useful to mitigate the identified risks substantially (52–54).

#### 4.1.4 Wishes regarding the content of information (RQ4)

**Figure 1:**
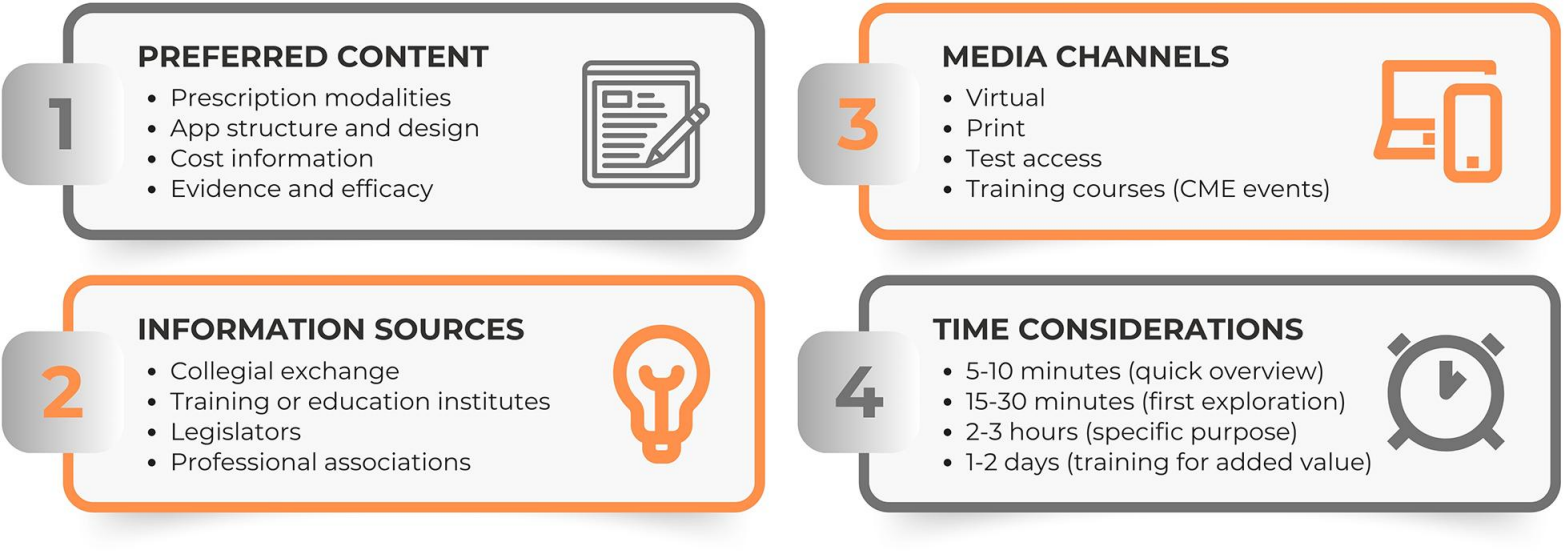
Examples for participants' information wishes: important content **(1)**, reliable sources **(2)**, preferred media channels **(3)**, time considerations **(4)**.

Regarding content information, our findings indicate that our participants value accessible evidence on the effectiveness, details about data privacy, and the costs of apps (see the illustrated summary in Figure 1). Other studies have also identified skepticism about the effectiveness of DiGA, concerns regarding adequate data protection, and reservations about their reimbursement (29, 48, 49, 51). In our interviews, we identified several important usability features, such as platform availability, language options, and accessibility settings. Berardi et al. (29) conducted a qualitative systematic review that highlighted similar factors, such as appealing design, ease of use, perceived usefulness, and portability of the device that the intervention offers, as major facilitators of implementing digital technologies in mental health systems. Additionally, psychotherapists requested clear information on indications, contraindications, and patient demographics, ideally presented in a tabular format, along with clarifying the prescription procedure. In their rapid review, Kreuzenbeck et al. (49) identified technical issues in prescribing DiGA. Similarly, Nogueira-Leite et al. (55) recognized perceived challenges with prescription in their mixed-methods study, while Seitz et al. (48) revealed concerns about interoperability with practice software for medical and psychological psychotherapists using an online survey. Quantitative research is required to confirm the key aspects for determining optimal information strategies for psychotherapists.

#### 4.1.5 Wishes regarding the delivery mode of information, e.g., by which sources and via media channels (RQ5)

To enhance the knowledge of psychotherapists regarding DiGA or DMHIs, it is essential to understand how therapists want to be informed. From their point of view, which are reliable information sources? Which are the preferred media channels?

Our interviews revealed that participants had differing opinions on reliable information sources, such as professional associations or manufacturers' websites (see the illustrated summary in Figure 1). They expressed a desire for sources that are neutral, relatively objective, authentic, and reliable. Specifically, they are looking for evidence-based content that is well-founded. This desire aligns with current research on what defines trustworthy sources, which identifies key characteristics such as competence in the relevant domain, sincerity, credibility, plausibility, and evidential support to ensure the content is sound (56, 57). Some mentioned sources, such as collegial exchange or patient feedback, are also according to research findings, indicating that reliability can be derived from recommendations or direct experience (56).

The media channels mentioned varied widely, including virtual or digital to printed, and postal formats. Participants thought newsletters and training courses were as valuable as online searches (see the illustrated summary in Figure 1). This diversity reflects findings from our earlier studies involving medical and psychology students (42, 58). To effectively address the information gap and enhance therapists' knowledge about DiGA (55, 59), it is essential to provide a broad range of access methods. Additionally, due to a lack of familiarity and possibilities (48), participants noted that test access to better determine the perceived usefulness of certain DiGA might ease their adoption (48, 60, 61).

Future studies should utilize methods such as cognitive interviews, focus groups, or quantitative surveys to explore a broader sample of psychotherapists, particularly those with less DiGA experience than those in our sample. This is to optimize information strategies for identifying the most suitable delivery modes across various scenarios and therapist groups in clinical practices. For example, this could include comparing in-person CME workshops to online courses, as well as evaluating test access or the use of printed material, all within specific frameworks (62).

#### 4.1.6 Additional topics

In our study, the willingness to invest time and effort to enhance knowledge on DiGA varied significantly among participants, as did their interest in paying for training (see the illustrated summary in Figure 1). While it is understood that education and training play a crucial role in facilitating the use of technologies (29), further exploration is needed to identify acceptable time and cost modalities.

As in our supplementary issue, participants gave insights into their attitudes toward AI, showing great interest. In line with recent studies, our participants expressed enthusiasm for AI tools in their field, while also expressing concerns about data security or ethical implications (63). To ensure ethical AI use in mental health contexts, future studies should investigate targeted training and regulatory measures to improve AI literacy (64).

#### 4.1.7 Overview of drivers and barriers for prescribing DiGA

Our findings on influencing factors can be divided into the prescription in general, the information about the apps, and therapeutic issues. According to participants, the lack of routine hinders DiGA prescribing, while familiarity with the process supports it (46). Providing training or better information may be beneficial to increase the uptake of DiGA (65). In a mixed-methods analysis of GPs, Weik et al. (59) emphasized several adoption barriers, including high workload, time constraints, insufficient information, and technical concerns. They suggested strategies for improvement, categorized as follows: developmental-related (e.g., better software integration), awareness-related (e.g., information from medical associations or colleagues), knowledge-related (e.g., details on benefits and reimbursement), implementation-related (e.g., reliable training materials), policy-related (e.g., simplified regulations and medico-legal guidelines). To successfully implement DMHIs in the USA, Zhao et al. (66) identified the need for special training, sample scripts, tutorials, or concise information sheets, which was among the requests expressed by our participants. Therefore, appropriate information material should be developed and tailored based on individual needs.

To situate these results in a broader context of adoption and implementation frameworks, they are mapped onto the identified drivers and barriers of the Updated Consolidated Framework of Implementation Research (CFIR) (67, 68). Within the CFIR's *Intervention Domain*, some constructs correspond closely to the information needs and wishes regarding DiGA features that psychotherapists in our study context indicated (e.g., *Source*: developer background, *Evidence-Base*: evidence and efficacy, *Adaptability*: app structure and design, *Trialability*: test access, *Cost*: cost information). However, in our study, we did not ask participants for their intervention preferences but for their wishes regarding information on DiGA for their practice. The CFIR also emphasizes the importance of *Access to Knowledge & Information* (e.g., guidance, training, or CME) for translating knowledge into actual, informed, and routine prescribing. Consistent with this, participants in our study reported scarce prior knowledge of DiGA, despite their overall positive attitudes and the fact that their profession had been authorized to prescribe DiGA for 4 years at the time of data collection. Linking these empirical findings to the well-established CFIR underscores that successful long-term adoption of DiGA requires multilevel alignment, tailored training for therapists, and structured support mechanisms (69–71).

### 4.2 Strengths and limitations of the study

One strength of this study is that we managed to reach a relatively diverse group of participants. Our interviews included psychological psychotherapists with varying levels of work experience, ranging from 4 to 21 years, and differing educational backgrounds. Notably, one psychotherapist expressed no interest in prescribing DiGA. At the same time, our sample distribution, in terms of gender and age, closely aligns with the population of psychotherapists in Germany (72). We are among the first to release specific qualitative study results for this target group in fall 2024, providing current data after 4 years of real-world experience since the implementation of the DiGA registry of the BfARM. Our study also benefits from the diverse backgrounds of our research team, which was mixed in terms of genders, disciplines (e.g., psychology, medicine, economics, public health), age, as well as professional and academic experiences. This feature likely broadens the range of views on the topic during the conceptual development and contextualization of our findings into a broader healthcare perspective.

When interpreting the findings, it is important to consider the following limitations. Selection bias may have occurred due to the convenient recruitment of participants, who were accepted in chronological order. Regarding issues related to self-selection, it should be considered that, especially in the field of DMHIs, participants with overall positive attitudes or interest in the topic appear to be more likely to participate in such studies (73–75). Using purposive sampling—focusing on factors such as experience, education, and location—could have provided a greater diversity of participants. Another relevant limitation might be that two-thirds of our participants had already been prescribed a DiGA, and all were aware of DiGA or DMHIs. It is unclear whether this high level of awareness results from the 4 years of DiGA implementation at the time of the interviews or if it is influenced by recruitment-related bias, with participants being particularly eager to discuss this topic (e.g., via LinkedIn as one recruitment channel, where we also promoted the study through our personal connections and postings).

The predominantly positive attitude towards DiGA that emerged in our analysis may not fully represent the views of psychotherapists who are less digitally engaged (e.g., older therapists, infrequent users of digital tools, or with limited digital health experience) (34). It may also under-represent therapists who do not use a behavioral (CBT) approach and are likely to be skeptical of DiGA, since CBT is the therapeutic modality most used in the currently listed DiGA (76). Therefore, the transferability of our findings to these less digitally engaged or non-CBT therapists should be interpreted with caution. Future studies should sample participants based on a range of experience with DiGA and digital literacy, including a larger group of psychotherapists unaware of these interventions, as this could provide a more comprehensive perspective. To obtain even broader and generalizable results, future studies should also include therapists with varying attitudes regarding DiGA, psychotherapists in training, and therapists employing different treatment approaches, such as analytical, systemic, or psychodynamic therapy. Finally, social desirability may have influenced the responses by the surveyed psychotherapists.

### 4.3 Implications for practice and future research

Future studies should focus on determining tailored information that, for example, addresses the barriers faced by psychotherapists to facilitate the possible adoption of DiGA (77). Likewise, further research is needed to evaluate special core competencies, such as integrating DiGA or DMHIs into the workflow, for determining useful training to support psychotherapists' perceptions (78, 79).

Recent studies suggest that experienced psychotherapists are generally more supportive of the potential benefits of DMHIs than non-experienced ones (80). Additionally, they are less likely to agree with the potential drawbacks associated with these interventions. It would be valuable to investigate whether increased experience with DiGA correlates with a more favorable perspective of prescribing them. Future research should examine confounding variables, such as age, gender, and workplace (rural/urban), which accounted for only a small amount of variance.

Future research should have more focus on evaluating psychotherapists' experience prescribing DiGA, as our study did not quantify how often they prescribe DiGA nor specifically ask how they have benefited patients. Our findings indicate that increased knowledge may lead to greater acceptance and adoption of DiGA, suggesting that tailored information is essential. With more knowledge and experience, psychotherapists may be more inclined to consider DiGA as a support or supplementary therapy, as we learn from other disciplines (81–83). Additionally, further research is required to determine the level of experience necessary to facilitate the prescription process for psychological psychotherapists in Germany.

In our sample, two-thirds reported already having prescribed at least one DiGA. However, we cannot be certain that all participants were fully familiar with the prescribing process of DiGA. Previous studies show that psychotherapists in Germany tend to have limited knowledge about the prescription procedure (30, 48). It may be beneficial to investigate the act of prescribing itself as a relatively new situation for the psychological psychotherapists in Germany to better understand potential concerns.

As the experience in our target group increases each year, the scope may not only focus on those who are unaware of DiGA, but also on informed decision-making regarding specific aspects (e.g., contraindications) and on how to stay updated. Recruiting psychotherapists who are reluctant to prescribe DiGA or are generally very skeptical concerning the usefulness of DMHIs would be interesting to complement the current findings in an upcoming study, as this would be out of scope for the current mixed-methods study on information needs and preferences among health professionals.

Particular findings from this formative qualitative research on the information needs and content wishes will be used to generate an experimental design for a DCE approach (84).

## 5 Conclusion and recommendations

From the perspective of surveyed psychological psychotherapists, DiGA appear to be a valuable, complementary form of therapy for mental health issues. At the same time, current concerns (e.g., managing upcoming patients' crises effectively, adequately addressing complex disorders) and uncertainties (e.g., about potentially facing recourse claims, about the prescription procedure) need to be considered to foster informed decisions concerning the adoption of DiGA in mental healthcare. Important wishes are comprehensive content information (e.g., on evidence, cost information, app structure), trustworthy sources (e.g., collegial exchange, professional associations), and appropriate media channels (e.g., test access, training courses, digital or in-person). Finally, as DMHIs, including DTx or DiGA, are still not a fixed part of the training of psychological psychotherapists in Germany, our study may offer several insights on how to modernize the therapeutic training in terms of digital health for psychotherapists, as well as the curriculum for CME workshops.

## Data Availability

The datasets presented in this article are not readily available because data cannot be shared publicly, as the transcripts may contain sensitive information. The data may be obtained from the corresponding author upon reasonable request, provided that legal frameworks are not violated and that responsibilities and confidentiality have been clarified. Requests to access the datasets should be directed to karin.panitz@uni-duesseldorf.de.

## Footnotes

^1^In the German statutory health insurance system, recourse claims (*Regressforderungen*) are retrospective reimbursement demands arising from economic efficiency audits (*Wirtschaftlichkeitsprüfungen*). When a physician’s prescribed services (e.g., medication, physiotherapy, DiGA) exceed the allocated budget or lack sufficient medical justification, the German Association of Statutory Health Insurance Physicians may initiate an audit. Over-billed prescriptions are cancelled, and the physician must bear the resulting cost adjustments (see https://www.kbv.de/html/2949.php).
