# Supplementary material for: Perspectives on digital therapeutic prescribing: a qualitative study among German psychological psychotherapists
Source: Front Digit Health. 2026 Feb 9;8:1656614. doi: 10.3389/fdgth.2026.1656614 (PMC12927034; doi:10.3389/fdgth.2026.1656614)
Supplement: Supplementary file 1 [file Datasheet1.pdf]

Supplementary Material

Supplementary Table 1. COREQ Checklist.

No.	Item	Description	Section/Notes
Domain 1: Research team and reflexivity			
Personal characteristics			
1	Interviewer/ facilitator	Which author/s conducted the interview or focus group?	#2.3
2	Credentials	What were the researcher's credentials? *E.g. PhD, MD*	#2.4
3	Occupation	What was their occupation at the time of the study?	#2.4
4	Gender	Was the researcher male or female?	#2.3
5	Experience and training	What experience or training did the researcher(s) have?	#2.4
Relationship with participants			
6.	Relationship established	Was a relationship established prior to study commencement?	#2.3
7.	Participant knowledge of the interviewer	What did the participants know about the researcher? *E.g. personal goals, reasons for doing the research*	#2.3
8.	Interviewer characteristics	What characteristics were reported about the interviewer/facilitator? *e.g. Bias, assumptions, reasons and interests in the research topic*	#2.3
Domain 2: Study design			
Theoretical framework			
9.	Methodological orientation and Theory	What methodological orientation was stated to underpin the study? *E.g. grounded theory, discourse analysis, ethnography, phenomenology, content analysis*	#2.1, #2.3
Participant selection			
10.	Sampling	How were participants selected? *E.g. purposive, convenience, consecutive, snowball*	#2.2
11.	Method of approach	How were participants approached? *E.g. face-to-face, telephone, mail, email*	#2.2
12.	Sample size	How many participants were in the study?	#2.2, #3.1
13.	Non-participation	How many people refused to participate or dropped out? What were the reasons for this?	#2.2, Supple-ment. Figure 1
Setting			
14.	Settings of data collection	Where was the data collected? *E.g. home, clinic, workplace*	#2.3
15.	Presence of non-participants	Was anyone else present besides the participants and researchers?	#2.3
16.	Description of sample	What are the important characteristics of the sample? *E.g. demographic data, date*	#3.1, Table 2
Data collection			
17.	Interview guide	Were questions, prompts, guides provided by the authors? Was it pilot tested?	#2.3, Supple-ment. Table 2
18.	Repeat interviews	Were repeat interviews carried out? If yes, how many?	no
19.	Audio/visual recording	Did the research use audio or visual recording to collect the data?	#2.3
20.	Field notes	Were field notes made during and/or after the interview or focus group?	#2.3
21.	Duration	What was the duration of the interviews or focus group?	#3.1
22.	Data saturation	Was data saturation discussed?	#2.3

23.	Transcripts returned	Were transcripts returned to participants for comment and/or correction?	no
Domain 3: Analysis and findings			
Data analysis			
24.	Number of data coders	How many data coders coded the data?	#2.4
25.	Description of the coding tree	Did authors provide a description of the coding tree?	#2.4, Supple-ment Table 4
26.	Derivation of themes	Were themes identified in advance or derived from the data?	#2.4
27.	Software	What software, if applicable, was used to manage the data?	#2.3
28.	Participant checking	Did participants provide feedback on the findings?	#2.3
Reporting			
29.	Quotations presented	Were participant quotations presented to illustrate the themes/findings? Was each quotation identified? *E.g. Participant number*	#3, #3.2-3.6
30.	Data and findings consistent	Was there consistency between the data presented and the findings?	#3
31.	Clarity of major themes	Were major themes clearly presented in the findings?	#3
32.	Clarity of minor themes	Is there a description of diverse cases or discussion of minor themes?	#3

Supplementary Table 2. Brief background questionnaire (translated, German to English).

**Pseudonymization code** (to be filled in by the investigator)**:** _______________
**Personal details**
(1) How old are you at the time of the scheduled interview? _____________ years
(2) What is your gender?<br>☐ female ☐ male ☐ diverse/other
(3) Which degree program/s have you completed?<br>(multiple answers are possible)<br>☐ Human medicine ☐ Psychology<br>☐ Other, namely: _________________________________
(4) Have you completed specialist medical and/or psychotherapeutic training?<br>☐ Yes, in the specialist field: ___________________________________<br>☐ No, I am currently not in (further) training<br>☐ No, but I am currently in further training,<br>in the subject/subject area: ___________________________________
(5) In which federal state do you work as a specialist? ___________________________________
(6) How do you rate the location of your place of work?<br>☐ (rather) rural ☐ (rather) urban
(7) To what extent are you currently working?<br>☐ Yes, full-time,<br>☐ Yes, part-time,<br>☐ Yes, other, namely ____________________________<br>☐ No, comment (optional): _____________________<br>☐ No specification
(8) <u>If you are self-employed</u>: Do you have a statutory health insurance license?<br>☐ Yes ☐ No
(9) How many years of practice do you have? _____________ years (since graduation)

**Awareness of digital interventions for mental health purposes**
(10) Have you heard of specific digital health intervention for mental health (digital mental health interventions, DMHIs) for preventive purposes (e.g., stress reduction and/or treatment included before participating in the study?<br>☐ No<br>☐ Yes, namely in the area/s, optional answer: ______________________________<br>____
(11) If you answered "yes" to question 10:<br>Have you ever prescribed a digital health intervention (German: Digitale Gesundheitsanwendungen, DiGA)?<br>☐ No<br>☐ Yes, namely (e.g., name of the DiGA or indication/diagnostic group):<br>____
(12) Are you already advising patients resp. clients on DiGA or DMHIs?<br>☐ Yes, I have already given advice on this<br>☐ I am still uncertain (see question 13)<br>☐ No, not interested (commentary, optional): ______________________________
(13) Do you intend to advise your patients resp. clients on DiGA or DMHIs in the future?<br>☐ Yes, it is planned<br>☐ Yes, but I don't feel adequately prepared, yet (commentary, optional):<br>____

Commentary (optional):

____

____

____

____

Supplementary Table 3. Guideline for semi-structured interview (translated, German to English)

*** = Optional follow-up questions, e.g., if the conversation stalls

**Procedure/topics**	**Suggested questions**
**Demands and needs**	
**0 Introduction & presentation**	Introduction of interviewer, study objectives („*Determine information preferences to maybe design optimal strategies for professionals*“), procedure explanation (at the start of the interview: *Video off, start of recording, please do not provide any personal data, etc.* – end: *recording will be stopped, video on*), clarification of open questions<br>Brief explanation of the terms „*digital mental health intervention (DMHI)*: *All interventions that support health electronically … this includes so-called digital health applications (short: DiGA – "apps on prescription")*
**1 Possible introductory question(s)**<br>(personal questions in the questionnaire), survey of attitudes	How did you hear about our study? *(Note: avoid clearly assignable information, otherwise blacken).*<br>What do you associate with the term DMHI/DiGA? What chances or risks do you see (for your specialization, for patients (P)/clients (C))?<br>*** What is your general attitude towards the issue of prescribing DiGA or use of DMHIs as a general practitioner (GP)/psychotherapist (PT)?<br>*** What opportunities and risks do you associate with DMHIs/DiGA?
**2 Possible (follow-up) questions to the topics of *prior knowledge & familiarity with the topic***<br>(if necessary: Specific questions on the brief pre-interview questionnaire)	What do you already know about DMHIs/DiGA?<br>How well informed do you feel about DMHIs/DiGA, e.g., to advise P/C? Where do you think is room for improvement?<br>*** Which DMHIs or DiGA for mental health do you know (e.g., free available or prescribable apps)?<br>(*** How familiar are you with this?)<br>*** Do you know special DiGA („apps on prescription“)? What do you think of DiGA? Have you ever prescribed a DiGA? (If so: which one? If no: why not?) (Alternatively: What are arguments for or against prescribing DiGA?)
**3 Possible (follow-up) questions to the topics of *digital health in the professional career and environment***	(To what extend) Have you already received/discussed information (prescription, type of services, etc.) on DMHIs/DiGA (reminder: digital mental health interventions resp. digital health applications) in your professional career (studies, training, further education)?<br>To what extend has the topic of DMHIs/DiGA been dealt with in your working environment (e.g., clinic, internal training, etc.)?<br>*** How do your superiors and/or colleagues think about this? To what extend is there an exchange on the topic and/or on existing trainings to DMHIs/DiGA?<br>*** What do you think about receiving information about DMHIs/DiGA from other professionals or exchanging information (e.g., mentors, team meetings)?<br>What benefits/incentives would motivate you to take a closer look at DMHIs/DiGA?
**4 Possible (follow-up) questions to the topics of *experience with use (patients), general wishes/preferences for use***	Can you estimate the time required to prescribe a DiGA?<br>Is a prescription realistic in your work routine?<br>*** What would you need (additional material, etc.) to ease the process?<br>What would be your motivation for prescribing a DiGA?

	Which offers could be particularly relevant for your P/C?<br>For which indication would you prescribe a DiGA (reminder: app on prescription)? Would you exclude or favor certain P/C or age groups?<br>Do you know whether your P/C already used DMHIs/DiGA?<br>*** If you have already received feedback from P/C according to the use of DMHIs/DiGA: What may be facilitating or hindering factors for the use?<br>*** If you have already recommended or prescribed DiGA: What experiences did your P/C report? What is the general resonance?<br>**** How do you experience cooperation with other authorities concerning DiGA, e.g., cooperation with providers, health insurance companies, P/C?
**Wishes (preferences)**	
**5 Possible (follow-up) questions to the topics of *wishes/preferences concerning the search for (online) health information, and presentation modalities***<br>Time and effort	How do you proceed to obtain information on new health services (especially in the field of mental health)?<br>As a GP or PT, in which way/through which medium would you like to be informed about existing offers?<br>Which media (virtual vs. non-virtual) do you prefer here?<br>***Which media/information channels do you prefer to obtain relevant information quickly?<br>How much time are you willing to invest in the (first) search, for a first impression, for information on new offers or to check offers? (*note: this is about the first information/impression*)<br>How much are you willing to invest in further training on DMHIs/DiGA (time, effort, other costs)?
**6 Possible (follow-up) questions to the topics of *wishes/preferences for the content of information on DMHIs/DiGA***<br>(quality criteria, important attributes)	What information (in terms of content) is important to you, to make an informed decision about recommending or prescribing a DiGA?<br>➔ What would you like to know in advance? What influences your decision? (What quality features come to your mind?)<br>➔ Which types of such DMHIs/DiGA, for which indication, would be particularly helpful for your P/C and therefore important to know?<br>What information would you prevent/hinder recommending/prescribing a DiGA?<br>*** What role does data protection information play for you?<br>How important is information on costs or cost coverage (for advising your P/C)? How detailed do you wish this information to be?<br>*** What are the three most important contents of information materials from your point of view?
**7 Possible (follow-up) questions to the topics of *design and presentation wishes/preferences for information on DMHIs/DiGA***	What appeals to you in terms of design if you were about to inform on DMHIs/DiGA?<br>In what form should the information be prepared or communicated (e.g., rather text, videos, podcasts, texts with images, etc.; different media formats)?<br>➔ What scope ([text] length or video/podcast [duration]) do you prefer? (time, effort depending on media)<br>➔ How important is interactivity to you when acquiring information (e.g., workshop vs. lecture)<br>*** What do you not like? (e.g., something like visually overwhelming, overloaded websites, too much technical language)<br>How should the information be presented linguistically/formally? (if you want to inform yourself and if you want to refer P/C to information material)

**8 Possible (follow-up) questions to the topics of *trustworthiness, credibility & recommendations***	What source of information to you find trustworthy? Who is important to you as a source of information? (*on request, if there is no answer: examples such as health insurance companies, journals, university, media, colleagues, etc.)*<br>What exactly creates trust for you/makes information on apps credible? (maybe again asking whether DiGA directory is known)<br>*** How do you feel about quality seals/seals of approval/certifications for apps? Which ones do you know?<br>What do you think of testimonials (concerning information material)? Who should inform P/C? (e.g., GP/PT/other experts, other P/C …)<br>How important is information on scientific evidence to you, and how detailed should it be?<br>Who should provide the information (for professionals)? (e.g. [medical] associations, research institutions, department of health, etc.)
**Closing**	
**9 Possible (follow-up) questions to the topics of**<br>***Further wishes and suggestions*** *(open)*<br>***"Mircale question"***	Do you have any suggestions or are there any aspects that we have not yet discussed?<br>*** What would be the optimal DMHIs/DiGA training for you as a professional (GP/PT), for example, if you had unlimited resources (in relation to requests for information strategies)?<br>*** What would be the optimal DMHIs/DiGA offer for your P/C or special P/C groups, if you had unlimited resources (in relation to intervention requests)?
Additional, starting July 27, 2024: ***Attitudes toward artificial intelligence (AI)***	How important do you think it is for medical professionals to be informed about the latest AI developments?<br>Do you have any specific requests or ideas about how you would like to be informed most effectively about new developments in the field of AI?
**Farewell**	End of interview, thanks for participating, clarify open questions, information to process of the expense of compensation

Note. The guideline may change during the survey. This is common in qualitative research to react to unexpected content. *Abbreviations*. AI = Artificial intelligence, C = Client, DiGA = Digital therapeutics (German *Digitale Gesundheitsanwendungen*), DMHI = Digital mental health intervention, GP = General practitioner, P = Patient, PT = Psychotherapist
