# Supplementary material for: Perspectives on digital therapeutic prescribing: a qualitative study among German psychological psychotherapists
Source: Front Digit Health. 2026 Feb 9;8:1656614. doi: 10.3389/fdgth.2026.1656614 (PMC12927034; doi:10.3389/fdgth.2026.1656614)
Supplement: Supplementary file 2 [file Datasheet2.pdf]

Supplementary Material

Supplementary Figure 1. Recruitment Flow Diagram.

Based on PRISMA 2009 Flow Diagram, modified

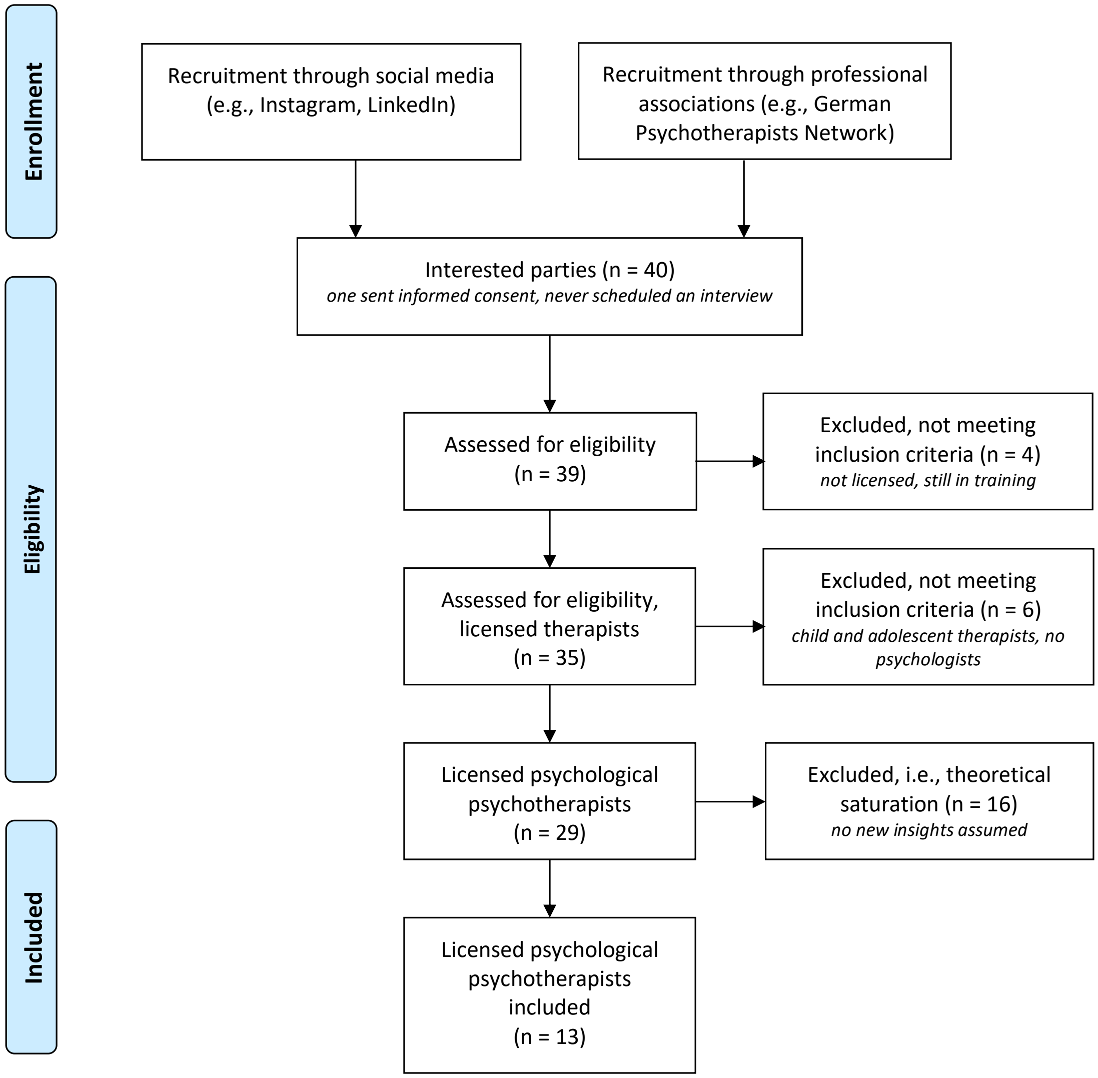

From: Moher D, Liberati A, Tetzlaff J, Altman DG, The PRISMA Group (2009). *P*referred *R*eporting *I*tems for *S*ystematic Reviews and *M*eta-*A*nalyses: The PRISMA Statement. PLoS Med 6(6): e1000097. doi:10.1371/journal.pmed1000097

For more information, visit www.prisma-statement.org.
