# Supplementary material for: Perspectives on digital therapeutic prescribing: a qualitative study among German psychological psychotherapists
Source: Front Digit Health. 2026 Feb 9;8:1656614. doi: 10.3389/fdgth.2026.1656614 (PMC12927034; doi:10.3389/fdgth.2026.1656614)
Supplement: Supplementary file 3 [file Datasheet3.pdf]

Supplementary Material

Supplementary Table 4. Excerpt from the coding scheme and anchor examples (translated, German to English)

**Level 1**	**Level 2 (Level 3)**	**Anchor examples** (quotes marked with *PT* for psychotherapist, the participants' *number*, and *gender*)
**RQ 1: What do psychotherapists in Germany already know about DiGA?**		
Prior knowledge & familiarity with the topic	Association with the term [d]	Generally, I associate it [DiGA, DMHI] with e-mental health techniques that have additional data processing, which should focus on various medical treatment areas, from respiratory tracts to mental health or smoking cessation. Above all, I associate it with modernization, that means usage via web or apps, in Google Android system or Apple system. (*PT1, male*)
	Current state of knowledge [i]	I think information offered by the BfArM [German Federal Institute for Drugs and Medical Devices] for example, are very, very plausible. And I think that the individual information of the particular DiGA in the directory are also well-structured and presented in order to compare them. At the same time, I do not feel very well informed when choosing individual DiGA, as there is a great abundance of different options, which is fantastic of course. (*PT7*, *female*)<br>Well, I know that DiGA exist, and I also know some areas of application that are also covered by the statutory health insurance. But overall, I feel rather poorly informed. (*PT13, female*)
	Previous information channels [d]<br>(level 3, i.e., collegial exchange, professional associations)	Yes, maybe in my intervision group [peer supervision], for example. We just had a discussion about that issue. Well, we are all rather younger therapists, so we might be even more motivated to use it. We are really interested in the topic. (*PT9, female*)<br>I recently attended the German Psychotherapy Conference, where I took part in an industry workshop on virtual reality glasses that are used in anxiety treatment for exposure therapy, for example. (*PT4, male*)
**RQ 2: Which experiences do psychotherapists have with recommending or prescribing DiGA?**		
Experience with use of DiGA	Previous prescription practice (behavior) [d]	So far, I have only done it selectively, and I am a little excited myself. (*PT8, male*)
	Effort and practicability of the prescription process [d]	I have to take care of it, but the organization doesn't take much time. I just have to issue the prescription and inform the patient of course. And it was important to me that it was somehow embedded [in the therapy]. I think it's absolutely necessary to combine it with my personal provision of information to patients. Somehow, I think therapy needs to be more than that. (…). You have to do some sort of follow-up at least, to see what has been implemented, what has worked, and that's what is time-consuming. But yes, issuing the prescription as such is quick, filling out the form, this takes ten or 15 minutes. (*PT12, female*)

	Feedback from patients and observed usage behavior of patients [i]	I usually can't really say much about that, because I've only prescribed it in the office hours so far. And I don't get to see these people more than once. (*PT5, female*)<br>Yes, well for the program addressing sleep disorder we have in fact received feedback. The patient said it was very descriptive which helped her with the information transfer, and that the structure actually worked out good for her. (*PT9, female*)
**RQ 3: Which attitudes do psychotherapists have toward recommending or prescribing DiGA?**		
General attitude, opportunities and risks concerning the topic	Need for information improvements (perceived information gaps) [d]	I just thought about this: I don't know if you could maybe prescribe a DiGA to patients who come to your office hours and who you have to send away trying to provide them with help on how to get a place in therapy. (...) In these cases, prescribing a DiGA would be great since they don't have such a place yet. (*PT2, female)*
	Incentives to look for more information or prescribe DiGA [d]	I would say that significantly better remuneration is necessary. This would be important to motivate many in the outpatient sector. And the specification of training regulations is essential, for example further education points or credits, so that especially older participants from the outpatient sector might be able to get credit points, because we always need to get these and everything should also be free of charge if possible. (*PT1, male*)
	Perceived benefits of DiGA [d]	In fact, I also see great opportunities, especially for patients who have a limited access to in-person psychotherapy or who might have inhibitions or difficulties in searching or finding therapy places. I think, especially in the rural area, to gain a foothold. And also, to boost the initial motivation and to cover aspects such as knowledge transfer. (*PT9, female*)<br>[…] to actually bridge waiting periods, in addition to ongoing psychotherapy or toward the end of therapy to prevent relapse, as an aftercare so to speak. (*PT9, female*)
	Perceived barriers of DiGA [d]	We see a lot of complex disorders and there's a risk that what is communicated might not be enough, perhaps. (…) Perhaps you don't look close enough in the first consultation sometimes. First consultations are comparatively well paid here, for psychiatrists as well. And you could be tempted to do that a lot, providing little therapeutic care to only prescribe apps, which then turn out to be not really suitable for many people. (*PT12, female*).<br>Or that people maybe even (…) rely on it too much and think, oh, then I don't need psychotherapy anymore, but that actually depends very much on the individual case. (*PT5, female*)
**RQ 4: What do psychotherapists want to know about DiGA (e.g., content of information)?**		
Content information wishes	Evidence and efficacy [d]	So, logically, I'd want the DiGA directory to include a meta-analysis that is kept up to date. I know that this is not possible. You'd just have to do it again every two or three years or simply work with updates. (*PT1, male*)<br>It needs to be certified. I don't always want to ask myself whether an app is thoughtfully realized from a professional perspective. I would assume that it is. (*PT12, female*)
	Data protection [d]	I don't want to search for information on server locations, on parties involved or on data storage. These are very relevant issues that need to be visible right away. (*PT9, female*)<br>I would assume that they're not involved in data fishing when they are certified and that other companies from the same sector wouldn't use health data elsewhere without data protection. (*PT3, female*)

	Cost information [d]	I think this is also interesting and relevant for the patients, because you can inform them about costs that are covered by their health insurance. And that is definitely valuable. (*PT6, female*)
	User-friendliness [i]	Above all, would it be possible to make them a bit more accessible with regard to age-specific, so gerontopsychiatric aspects or characteristics concerning learning disabilities. (*PT1, male*)
	App structure and design [i]	Perhaps you could briefly compare their contents. I have no idea, for example elements for behavioral activation, and (..) cognitive restructuring, but also the ABC scheme [by Albert Ellis[1]], depending on what is included in the app. (*PT10, female*)
	Other prescription-relevant factors [i]	Are there contraindications maybe? Which patient group is it aimed at and for whom is it suitable? (*PT13, female*)
	Prescription modalities [i]	What might be good, but I am not sure if it's already somehow provided in the accounting systems [practice software], I am not sure, but as far as I know, this number [pharmaceutical registration number] has to be included in the prescription for the app. And to be honest, I don't know if it's included in the systems. If not, I would appreciate an overview, if the correct number for the prescription would be listed. (*PT10, female*)
	Updates on certain DiGA [I]	Information on potential updates, of course. Because with an update, I might consider a DiGA, which I didn't like before (*PT1, male*)
	Developer/provider background [i]	Also, who developed it [the DiGA], and what's the technical background. (*PT12, female*)
**RQ 5: How do psychotherapists want to be informed about DiGA (e.g., by which sources and via which media channels)?**		
Presentation wishes	Information sources [d]<br>(level 3, i.e., training/ education institutes, legislators, associations)	How about further training in general also with credit points? I think it's a pity that there are only few informational events that are not organized by the developers themselves. There are almost no events providing broader or maybe also more objective information to familiarize yourself with the topic. (*PT7, female*)<br>I always say I'd like to have a lucid table, perhaps also provided by the Ministry of Health. (*PT1, male*)
	Media channels [d]<br>(level 3, i.e., virtual, print, newsletter)	I would in fact prefer online training. (*PT10, female*)<br>Journals and emails actually reach me very well, for example the email that informed me about this study. (*PT2, female*)

[1] Ellis' ABC Model for the development of behaviors and possible assessment through perception of A: Activating experience, B: Beliefs, C: Consequences; Reference Tiba, A. I. (2024). The grounded cognition foundation of the first cognitive model in cognitive behavior therapy: Implications for practice. Frontiers in Psychology, 15, 1364458. https://doi.org/10.3389/fpsyg.2024.1364458

Additional insights		
Time/cost considerations	Willingness to invest in information acquisition [d] (level 3, i.e., search, further training: time, further training: costs)	I would say it takes about two to three hours to initially search for an app that fits. (*PT13, female*)<br>Well, depending on the area, I'd invest a different amount of time. If I see an additional value for my therapeutic practice, I'd certainly invest more time. (…) Let's say, once a year I'd spend around two days solely on that topic to educate myself further. (*PT3, female*)<br>That depends – if it was for the job or an employer, I would expect the employer to finance it, so I wouldn't want to pay anything at all. If it was in the context of my self-employment, for example, as further training, then I would say, I'd pay 350 euros, if it would be interesting for me, and maybe about 100 euros for virtual training. (*PT13, female*)
Attitudes toward AI	Importance [d], type of information [i], channels [i]	I think that's very important. Because it's certainly something that is interesting for many. It also has something to do with your own professional future, for example to what extent AI might do tasks for you or contribute to your decision-making. (*PT4, male*)<br>I don't have any specific ideas, but I think that perhaps digital information would be very helpful, preferably free of charge, to continuously shed light on various aspects on a regular basis. For example, a serial like once a month or week where you have the opportunity to catch up on specific issues that are then presented. I think I would use that to some extent. Especially, if it's free of costs. (*PT8, male*)

Note. [d] deductively coded, [i] inductively coded. Level 3 codes are presented in parts. *Abbreviations*. AI = Artificial intelligence, BfArM = German Federal Institute for Drugs and Medical Devices (German *Bundesinstitut für Arzneimittel und Medizinprodukte*), DiGA = Digital therapeutics (German *Digitale Gesundheitsanwendungen*), PT = Psychotherapist, RQ = Research questions
