# Supplementary material for: Perspectives on digital therapeutic prescribing: a qualitative study among German psychological psychotherapists
Source: Front Digit Health. 2026 Feb 9;8:1656614. doi: 10.3389/fdgth.2026.1656614 (PMC12927034; doi:10.3389/fdgth.2026.1656614)
Supplement: Supplementary file 4 [file Datasheet4.pdf]

Supplementary Material

Supplementary Table 5. Self-reported awareness of DiGA for mental purposes or DMHIs among psychological psychotherapists (*N* = 13).

Predefined answers	*n* (%)
**Have you heard of specific DiGA for mental health or DMHIs before participating in the study?**	
Yes	13 (100.0)
**Have you ever prescribed a DiGA?**	
Yes	8 (61.5)
No	5 (38.5)
**Are you already advising clients on DiGA or DMHIs?**	
Yes, I have already given advice on this	10 (76.9)
I am still uncertain	2 (15.4)
No, not interested	1 (7.7)
**Do you intend to advise your clients on DiGA or DMHI in the future?**	
Yes, it is planned	8 (61.5)
Yes, but I don't feel adequately prepared, yet	4 (30.8)
Voluntary comments on "*Yes, but I don't feel adequately prepared, yet*"	Concern about abolishing their own job; Prescription regulations, evaluation/benefits, meaningfulness; Prescription options, possible applications, risks, and limits
No	1 (7.7)

Abbreviations. DiGA = Digital therapeutics (German *Digitale Gesundheitsanwendungen*); DMHIs = Digital mental health interventions.

Supplementary Table 6. Comparison of prescribers (n = 8) and non-prescribers (n = 5) regarding their attitudes toward prescribing DiGA (N = 13).

Prescribed DiGA: Yes (*n* = 8)	Prescribed DiGA: No (*n* = 5)
**Attitude topic: Need for information improvement.**	
	Comprehensive guideline to prescribe a DiGA (*PT1, male*)
	When to issue prescriptions (*PT2, female*)
How to integrate the process into routine practice (*PT9, female*)	
**Attitude topic: Incentives to look for more information or prescribe DiGA.**	
Improved financial compensation (*PT10, female*)	Improved financial compensation (*PT1, male; PT2, female*)
Compensation: CME points (*PT5, female*)	Compensation: CME points (*PT1, male; PT2, female*)
	Compensation: free training (*PT1, male*)
Seeing patients succeed through a DiGA (*PT8, male*)	
**Attitude topic: Perceived benefits of DiGA.**	
Bridging waiting periods (*PT6, female; PT10, female; PT12, female*)	Bridging waiting periods (*PT1, male; PT13, female*)
Serving as a supplement (*PT3, female; PT5, female; PT6, female; PT8, male*)	
Serving as partial substitute for face-to-face therapy (*PT5, female; PT6, female*)	
Improving initial motivation (*PT9, female*)	
Outsourcing therapy aspects, e.g., psychoeducation (*PT5, female*)	Outsourcing therapy aspects, e.g., psychoeducation (*PT4, male*)
Aiding aftercare (*PT6, female*)	
	Support after basic care (*PT1, male*)
Relapse prevention (*PT6, female*)	Relapse prevention (*PT4, male*)
Facilitate access to therapy (*PT9, female*)	
Usefulness of special journal functions (*PT8, male*)	Usefulness of special journal functions (*PT4, male*)
**Attitude topic: Perceived barriers of DiGA.**	
Lack of established routines (*PT9, female*)	
	Possible underestimation of psychotherapy's effectiveness (*PT4, male*)
Prevent patients from beginning person therapy (*PT5, female*)	
May not adequately address complex disorders (*PT3, female; PT5, female; PT12, female*)	
Potential to overwhelm patients (*PT6, female*)	

Unsuitable applications (*PT6, female; PT12, female*)	
	Insufficient personal support (*PT11, male; PT13, female*)
	Elimination of current practices, e.g., due to competition, lower costs (*PT2, female; PT11, male*)
Concerns on managing upcoming crises effectively (*PT9, female*)	Concerns on managing upcoming crises effectively (*PT11, male*)
	Potential recourse claims (*PT2, female*)

Note. The participant code is shown in brackets (*PT* for psychotherapist, the participant's *number*, and *gender*). *Abbreviations.* CME = Continuing Medical Education, DiGA = digital therapeutics (German *Digitale Gesundheitsanwendungen*).
